# Siglec-7 Mediates Immunomodulation by Colorectal Cancer-Associated *Fusobacterium nucleatum* ssp. *animalis*

**DOI:** 10.3389/fimmu.2021.744184

**Published:** 2021-10-01

**Authors:** Dimitra Lamprinaki, Pilar Garcia-Vello, Roberta Marchetti, Charlotte Hellmich, Kelli A. McCord, Kristian M. Bowles, Matthew S. Macauley, Alba Silipo, Cristina De Castro, Paul R. Crocker, Nathalie Juge

**Affiliations:** ^1^ Quadram Institute Bioscience, Norwich Research Park, Norwich, United Kingdom; ^2^ Department of Chemical Sciences, University of Naples Federico II, Naples, Italy; ^3^ Norfolk and Norwich University Hospitals, NHS Foundation Trust, Norwich, United Kingdom; ^4^ Departments of Chemistry, and Medical Microbiology and Immunology, University of Alberta, Edmonton, AB, Canada; ^5^ Norwich Medical School, University of East Anglia, Norwich, United Kingdom; ^6^ Department of Agricultural Sciences, University of Naples Federico II, Portici, Italy; ^7^ Division of Cell Signalling and Immunology, School of Life Sciences, University of Dundee, Dundee, United Kingdom

**Keywords:** *Fusobacterium nucleatum*, colorectal cancer, Siglec-7, outer membrane vesicle, innate immunity, lipopolysaccharide

## Abstract

*Fusobacterium nucleatum* is involved in the development of colorectal cancer (CRC) through innate immune cell modulation. However, the receptors of the interaction between *F. nucleatum* ssp. and immune cells remain largely undetermined. Here, we showed that *F. nucleatum* ssp. *animalis* interacts with Siglecs (sialic acid–binding immunoglobulin-like lectins) expressed on innate immune cells with highest binding to Siglec-7. Binding to Siglec-7 was also observed using *F. nucleatum*-derived outer membrane vesicles (OMVs) and lipopolysaccharide (LPS). *F. nucleatum* and its derived OMVs or LPS induced a pro-inflammatory profile in human monocyte-derived dendritic cells (moDCs) and a tumour associated profile in human monocyte-derived macrophages (moMϕs). Siglec-7 silencing in moDCs or CRISPR-cas9 Siglec-7-depletion of U-937 macrophage cells altered *F. nucleatum* induced cytokine but not marker expression. The molecular interaction between Siglec-7 and the LPS O-antigen purified from *F. nucleatum* ssp. *animalis* was further characterised by saturation transfer difference (STD) NMR spectroscopy, revealing novel ligands for Siglec-7. Together, these data support a new role for Siglec-7 in mediating immune modulation by *F. nucleatum* strains and their OMVs through recognition of LPS on the bacterial cell surface. This opens a new dimension in our understanding of how *F. nucleatum* promotes CRC progression through the generation of a pro-inflammatory environment and provides a molecular lead for the development of novel cancer therapeutic approaches targeting *F. nucleatum*-Siglec-7 interaction.

## Introduction

Colorectal cancer (CRC) is one of the most frequently diagnosed malignancies worldwide, accounting for 10% of all cancers and for approximately 20% of all cancer-related deaths in developed countries (1). While CRC incidence commonly appears in ages over 50 years old, recent years have shown an increased incidence in younger adults which may be associated to lifestyle factors (2, 3).

Tumours that arise at epithelial barrier surfaces of the body harbour extensive microbiota, and the importance of these microbes in CRC is now widely acknowledged. The enrichment of *Fusobacterium* spp. in CRC tissues, as revealed by whole genome sequencing, showed that the most abundant species is *Fusobacterium nucleatum* (4–6). Patients with *F. nucleatum* associated carcinoma have a shorter survival period (7). In addition, *F. nucleatum* appears to contribute to the chemoresistance of CRC (8–10). Among *F. nucleatum* subspecies, *F. nucleatum* ssp. *animalis* is most predominant in CRC specimens (11).

*F. nucleatum* potentiates intestinal tumorigenesis mainly by recruitment of tumour infiltrating immune cells, in particular myeloid-derived immune cells such as tumour associated macrophages (TAMs), myeloid-derived suppressor cells (MDSCs), dendritic cells (DCs), tumour associated neutrophils (TANs) (11–13), and inhibits human T-cell response (12, 14, 15), leading to colorectal neoplasia progression. A high abundance of *F. nucleatum* in CRC tissues is associated with increased nuclear factor kappa B (NFκB) activation and induction of a pro-inflammatory profile (12). Well-characterised virulence factors of *F. nucleatum* such as membrane proteins FadA or Fap2 are involved in the binding of *F. nucleatum* to colon cancer cells inducing oncogenic response (16, 17). In the epithelium, *F. nucleatum* induces the expression of cell signalling proteins (cytokines), such as tumour necrosis factor (TNF)-α and interleukin (IL)-8 in addition to epithelial-mesenchymal transition (18). Recently, the structures of O-chain polysaccharides (O-antigens) of the LPS from *F. nucleatum* strains ATCC 23726 (ssp. *nucleatum*) (19), ATCC 25586 (ssp. *nucleatum*) (20), ATCC 10953 (ssp. *polymorphum*) (21), ATCC 12230 (22), MJR 7757 B (23) and ATCC 51191 (*ssp. animalis*) (24) have been elucidated, showing strain-specific differences in the trisaccharide repeat unit containing either sialic acid/N-acetylneuraminic acid (Neu5Ac) (21), fusaminic acid (20) or monosaccharides other than nonulosonic acid residues (24). However, the mechanisms underpinning the interaction of *F. nucleatum* with immune cells remain undefined.

Immune cells express a large variety of glycan-binding receptors or lectins, which sense and respond to changes in the glycan signature of their environment leading to the activation or inhibition of immune processes (25). Siglecs (sialic acid–binding immunoglobulin-like lectins) are a large family of lectins found on innate immune cells and tumour-infiltrating T cells, which inhibit immune activation after sensing sialic acid-containing glycans (26, 27). Individual family members exhibit preferences for sialosides of various linkages to underlying glycan motifs, but many of the physiological ligands, glycoproteins or glycolipids, they interact with remain largely unknown (28, 29). The expression of Siglecs on immune cells is cell type dependent (30). Siglecs are transmembrane proteins, which possess an extracellular portion characterized by a V-set immunoglobulin-like domain, containing the carbohydrate recognition domain (CRD), and one or more C2-set immunoglobulin-like domains. The majority of Siglecs possess immunoreceptor tyrosine-based inhibitory motifs (ITIMs) in their intracellular domain (26). Siglec ligands can be presented on the cell on which the Siglec is expressed (*cis* ligands), or on glycans in the extracellular matrix of other cells (*trans* ligands) (28). Although the CRDs of most Siglecs have some specificities towards certain sialylated structures, several Siglecs have a broad and overlapping ligand specificity (28). These glyco-immune checkpoints have been proposed as new targets for cancer immunotherapy (31–33). The working hypothesis on the role played by Siglecs in cancer is that immune cells expressing Siglecs are inhibited upon binding to their ligands on cancer cells. Indeed, enzymatic removal of sialic acids from cancer cell surfaces was shown to enhance immune cell-mediated clearance of those cells through loss of Siglec-7 and Siglec-9 binding in *cis* (34) although the range of physiological ligands of Siglec-7 and Siglec-9 remain to be identified (35). Recently, a genome-wide CRISPR screens revealed the glycoprotein CD43 expressed on leukemia cells as a highly specific ligand for Siglec-7 and blocking the interaction relieved Siglec-7-mediated inhibition of immune killing activity (33). In addition, the tumour immune-suppressive effect of Siglec-7 was recently demonstrated *in vivo* (36), further supporting the proposed role of Siglec-7 as an immune checkpoint receptor.

Several clinically relevant pathogens have evolved mechanisms of molecular mimicry by displaying sialylated structures on their surface to overcome the *cis* interactions of Siglecs on the surface of immune cells. For example, *Campylobacter jejuni* strains can interact with Siglec-7 and sialoadhesin (Siglec-1) *via* their lipooligosaccharides (37) and to Siglec-10 *via* a sialic acid-like molecule, pseudaminic acid present in the flagella (38), while Siglec-7 showed sialic acid-independent binding to β-protein expressed on Group B Streptococcus surface (39). Additionally, human Siglecs have evolved to recognise non-Neu5Ac ligands present on external stimuli such as microbes (40). In this work we hypothesised that Siglecs may be involved in the recognition of *F. nucleatum* strains by immune cells, contributing to the tumorigenesis of these strains in CRC.

## Materials and Methods

### Materials

All reagents were purchased from Sigma unless otherwise stated. Recombinant Siglecs, human Siglec-3, Siglec-5, Siglec-7, Siglec-9 and -10 and CHO-expressing Siglec-7-Fc (CHO-Siglec-7-Fc) cell line were a kind gift from Prof. Paul Crocker (University of Dundee). Recombinant Siglec-7-Fc was also obtained commercially (R&D Systems).

### Bacteria Growth, Preparation and Quantification

*F. nucleatum* ssp. *animalis* ATCC 51191 isolated from clinical samples was obtained from ATCC in partnership with LGC standards ltd. *F. nucleatum* was cultured in tryptic soy broth media (Becton Dickinson) supplemented with 5 μg/ml hemin (Sigma) and 1 μg/ml menadione (Sigma). For binding and human cell co-culture experiments, bacteria were centrifuged at 15,000 x g for 5 min, and the cells were fixed with 4% paraformaldehyde (PFA) (Electron Microscopy Sciences/CN Technical Services ltd) for 45 min at room temperature (RT), in the dark, followed by two washes in Dulbecco’s Phosphate-buffered Saline (DPBS) (Lonza).

For bacteria de-sialylation, 10^7^ cells were treated with 20 U of sialidase α2-3,6,8,9 neuraminidase A (NEB) in 1X GlycoBuffer I (NEB) or control treated in 1X GlycoBuffer I alone, overnight (o/n) at 37°C.

Bacteria were quantified by spectroscopy with OD_600nm_ of 1 corresponding to 10^9^ cells/ml or by imaging flow cytometry (Amnis ImageStream^x^ Mk II) (as described below).

### LPS Extraction

*F. nucleatum* ATCC 51191 bacterial cells were harvested by centrifugation, lyophilised, and extracted by the hot phenol/water method, as previously described (41). Each phase was dialysed against distilled water to remove the phenol, freeze-dried, and analysed by 12% sodium dodecyl sulphate polyacrylamide gel electrophoresis (SDS-PAGE). After the water/phenol extraction, *F. nucleatum* LPS extracted from was detected in the water phase by silver nitrate staining (42). The phases containing LPS were further purified by enzymatic digestion (DNAse, RNAse and proteinase K) as previously described (41), followed by centrifugation at 6,000 rpm for 30 min at 4°C and ultracentrifugation at 30,000 rpm for 4 h at 4°C. To separate the O-antigen (OPS) and lipid A domains, LPS were mild acid hydrolysed by acetic acid 1% (100°C, 2-3 h). The OPS domain of the strains containing ulosonic residues was further partial depolymerised. The solution was centrifuged and the supernatants were freeze-dried and further purified by gel filtration chromatography.

### *F. nucleatum* OMV Purification and Characterisation

*F. nucleatum* derived OMVs were collected from the cell culture supernatant, as described previously by Liu et al. (43) with some modifications. Briefly, *F. nucleatum* ssp. cells were cultured until reaching OD_600nm_ of 0.7-1.2. Cells were centrifuged at 8,500 x g for 15 min at 4°C. The supernatant was collected, and vacuum filtered using 0.22 μm membrane. The filtered supernatant was concentrated by spin-filtration using 100,000 molecular weight cut-off filter unit (Sartorius). OMVs were recovered from the filter using sterile DPBS and further purified by density gradient ultra-centrifugation. For the gradient, Optiprep media (60% w/v, Sigma) was diluted in 0.85% w/v NaCl and 10 mM tricine-NaOH pH 7.4 solution to make 35%, 30%, 25% and 20% density solutions. The OMVs were mixed with 40% Optiprep solution and placed at the bottom of a 13.2 ml Ultra-clear tube (Beckman Coulter) and Optiprep (2 ml) was added subsequently by density-decreasing order. The preparation was ultracentrifuged at 135,000 x g for 16 h at 4°C with minimum acceleration and deceleration using a SW41 Ti rotor (Beckman Coulter). From the top to the bottom, 1 ml fractions were collected and analysed by SDS-PAGE in 4–15% Mini-PROTEAN^®^ TGX™ Precast Protein Gel (BIO-RAD). The OMV-containing fractions were diluted with sterile DPBS and ultracentrifuged at 200,500 x g for 2 h at 4°C using a Type 45 Ti rotor (Beckman Coulter). OMVs were resuspended in sterile DPBS and then filtered using a 0.22 μm membrane.

Purified OMVs were quantified and measured for their particle size using a NanoSight LM12 (Malvern Panalytical). Briefly, the samples were diluted 100 times in 1 ml DPBS and loaded onto the instrument’s chamber by a syringe and the sample were slowly released. The considered particle size of each OMV sample were the mean of triplicates. Instrument settings used: camera shutter 1035, camera gain 680, capture duration 60 sec.

### Semi-Quantitative Analysis of LPS in OMVs by Gas Chromatography-Mass Spectrometry (GC-MS)

The content of LPS in *F. nucleatum*-derived OMVs was evaluated by analysing the fatty acids content. Based on the chemical structure of the lipid A component of *F. nucleatum* LPS in the bacteria (44), C14:0 (or myristic acid) was considered as the reporter group for LPS, while C16:0 (palmitic acid) and C18:0 (stearic acid) were considered the reporters for the phospholipids.

*F. nucleatum*-derived OMVs (1 mg) were treated with HCl/MeOH (1 ml, 1.25 M, 80°C, 16 h) and lipids, derivatised as methylesters, were extracted with hexane (41). This analysis estimated the amount of each fatty acid (C14:0, C16:0 and C18:0) by correcting the areas of the corresponding peaks with a response factor, made by using an array of standard solutions and by setting C16:0 as internal standard. Areas were correlated by a linear regression. The methanol layer after extraction with hexane, was used to countercheck the data from lipid analysis, by verifying the presence of 3-deoxy-2-keto-D-*manno*-octulosonic acid and L-*glycero*-D-*manno*-heptose, both markers of the LPS molecules. Identification of the fatty acids or the monosaccharide constituents, was performed by comparing the retention time and the fragmentation pattern of each peak to a relevant standard.

All chemical derivatives were analysed by using a Gas Chromatography-Mass Spectrometry (GC-MS) Agilent Technologies 7820A (Santa Clara, CA, USA) equipped with a mass selective detector 5977B and a HP-5ms capillary column Agilent, Italy (30 m x 0.25 mm i.d., 0.25 μm as film thickness, flow rate 1 ml/min, He as carrier gas). Electron impact mass spectra were recorded with ionisation energy of 70 eV and an ionising current of 0.2 mA. The temperature program used was: 150°C for 5 min, 150 up to 300°C at 10°C/min, 300°C for 12 min.

### Expression and Purification of Recombinant Siglec-Fc Proteins

CHO-Siglec-7-Fc cells were cultured in Glasgow Modified Essential Medium (GMEM) without L-glutamine media (Sigma) supplemented with 10% fetal bovine serum (FBS) (Thermo Scientific Gibco), 100 U/ml penicillin and 100 μg/ml streptomycin (Lonza) and 50X GS supplements (Sigma).

Adherent CHO-Siglec-7-Fc cells (80-90% confluence) were washed twice with Dulbecco’s phosphate-buffered saline (DPBS) (Thermo Scientific Gibco) and protein expression was induced by culturing the cells with GMEM without L-glutamine media (Sigma) supplemented with 200X FetalClone II (Thermo Fisher Scientific), 100 U/ml penicillin and 100 μg/ml streptomycin (Lonza), 50X GS supplements (Sigma) and 100 μg/ml MSX (Sigma). After 4 days, the supernatant was collected for Siglec-7-Fc purification.

Siglec-7-Fc purification was carried out using gravity-flow column (BIO-RAD) packed with protein A-Sepharose (Sigma) washed with DPBS. The harvested CHO supernatant was added to the column and the column washed with DPBS. To elute Siglec-7-Fc, a solution of 0.1 M glycine, pH 3 was added to the column and fractions (0.5 ml) were collected in 1 M Tris, pH 8 (for neutralisation). The protein concentration in fractions was quantified by Nanodrop (Thermo Fisher Scientific).

### Binding Assays

For the flow cytometry binding assays between *F. nucleatum* ssp. animalis ATCC 51191 and recombinant Siglecs (Siglec-3, Siglec-5, Siglec-7, Siglec-9 and -10), bacteria (10^7^ cells) were incubated with the pre-complex of recombinant Siglec-Fc (4 μg/ml) and mouse α-Fc-PE Ab (1 μg/ml) (R&D Systems) in DPBS for 1 h at 37°C. Following centrifugation at 14,000 x g for 4 min, bacterial cells were washed with DPBS and analysed by Fortessa (BD Biosciences). For the inhibition assays, Siglec-7-Fc and a-Fc-phycoerythrin (PE) Ab pre-complex was first incubated with disialoganglioside with three glycosyl groups GD3 (Sigma) at 50 μg/ml for 30 min at 4°C. For the flow cytometry binding assays between *F. nucleatum* ssp. and human cells, U-937 (WT or Siglec-7^-/-^) cells were first stained with 10,000X cell trace violet (CTV) (Thermo Fisher Scientific) for 15 min at RT and *F. nucleatum* ssp. were stained with 10 μg/ml of fluorescein isothiocyanate (FITC) (Sigma). Following two washes with DPBS, U-937-CTV (5 x 10^5^ cells) were incubated with *F. nucleatum*-FITC (5 x 10^6^ cells) for 1 h at 4°C. Cells were washed with FACS buffer (HBSS containing 0.01% bovine serum albumin (BSA), and 2 mM EDTA), centrifuged at 510 x g for 3 min and analysed using Fortessa (Threshold of FSC parameter set to 1000). Flow cytometry data were processed in FlowJo (TreeStar) software.

For the ELISA-based binding assays, bacteria (10^7^ cells) or bacteria-derivatives (10 μg/ml LPS or 10^8^ OMV particles) in DPBS solution were coated in a 96-well plate, o/n at 4°C. Following a washing step with 0.05% tween in PBS (washing buffer) the plate was incubated with 1% BSA for 1 h at RT. Followed by 3 times washing the plates were incubated with pre-complexed Siglec-Fc and α-Fc-HRP for 2 h at RT. Briefly, Siglec-Fc protein (4 μg/ml) was incubated with 50,000X α-human-Fc-HRP (Abcam) for 1 h at RT. Following 3 washes with washing buffer, the plate was incubated with 3,3′,5,5′-tetramethylbenzidine (TMB) (Biolegend) until colour development. Colour development was stopped by the addition of 2 N H_2_SO_4_ and the absorbance was measured at 450 nm with reference at 570 nm. Data were analysed in GraphPad Prism 6.

### STD NMR Analysis

Spectra were acquired on a Bruker 600 MHz AVANCE NEO equipped with a cryo probe and analysed using the TOPSPIN 4.1.0 software. The partial depolymerised OPS derived from *F. nucleatum* ssp. LPS were prepared in deuterated PBS buffer (20 mM PBS, NaCl 150mM, pH= 7.4), using protein-ligand ratios varying from 1: 20 to 1: 80 with 15 µM of Siglec-7-Fc protein. STD NMR experiments were acquired at 298 K with 32 k data points and zero-filled up to 64 k data points prior to processing. The Siglec-7-Fc resonances were saturated applying 40 Gauss pulses with a length of 50 ms, setting the on-resonance pulse at aromatic region (7.5/6-5 ppm) and the off-resonance pulse frequency at 100 ppm. Under these experimental conditions, very low residual signals were observed in some of the STD NMR spectra for the ligands in the free state which were taken into account during data processing. To suppress the water signal, an excitation sculpting with gradient pulses (esgp) was applied and to reduce the NMR signals of Siglec-7-Fc, a spin-lock filter (20 ms) was used.

### Culture of Human Primary Immune Cells and U-937 Monocytic Cell Line

Human peripheral blood was obtained from haemochromatosis patients undergoing a therapeutic venesection at the Norfolk and Norwich University Hospital (Norwich, UK). Blood collection in this study was approved by the Faculty of Medicine and Health Sciences Research Ethics Committee REC reference number 2013/2014 -14HT (University of East Anglia).

For monocyte-derived dendritic cell (moDC) and macrophage (moMϕ) generation, peripheral blood mononuclear cells (PBMCs) were isolated from whole blood following centrifugation using Ficoll-Paque gradient media (GE Healthcare). Monocytes (CD14+ cells) were isolated from PBMCs using CD14 positive selection microbeads (StemCell technologies) according to the manufacturer’s instructions. Freshly isolated CD14+ monocytes (10^6^ cells/ml) were cultured in Mercedes medium (RPMI 1640 medium (Lonza) supplemented with 25 mM HEPES, 10% FBS (Thermo Scientific Gibco), 55 μM 2-mercaptoethanol, 100 U/ml penicillin and 100 μg/ml streptomycin (Lonza), 2 mM glutamine (Lonza), 1 mM non-essential amino acids (Lonza) and 1 mM sodium pyruvate (Lonza), were incubated with granulocyte-macrophage colony-stimulating factor (GM-CSF) and IL-4 (PeproTech) (25 ng/ml) for differentiation of monocytes to moDCs or with macrophage colony-stimulating factor (M-CSF) (PeproTech) (25 ng/ml) for differentiation of monocytes to moMϕs. The cells were incubated for 7 days at 37°C, with addition of the above cytokines on day 3, as previously described (45).

For U-937 differentiation, U-937 (5 x 10^5^ cells/ml) cultured in RPMI 1640 medium (Lonza) supplemented with 25 mM HEPES, 10% FBS (Thermo Scientific Gibco), 55 μM 2-mercaptoethanol, 100 U/ml penicillin and 100 μg/ml streptomycin (Lonza), 2 mM glutamine (Lonza) and 1 mM sodium pyruvate (Lonza) were differentiated with 100 ng/ml phorbol 12-myristate 13-acetate (PMA) (Sigma) for 28 h at 37°C, as previously described (46). Adherent cells were detached by PBS-EDTA (Lonza) and scraping and collected for functional assays.

### Generation of Siglec-7^-/-^ U-937 Cells by CRISPR-Cas9

CRISPR RNA (crRNA) was designed to target human Siglec-7 (CATGCCCTCTTGCACGGTCA, IDT) in U-937 cells (ATCC^®^ CRL-1593.2™). guide RNA (gRNA) (1 μM crRNA, 1 μM ATTO-550 labeled tracrRNA (IDT)) was boiled at 95°C for 5 min. A solution of 20 pmol gRNA, 20 pmol Cas9 nuclease (IDT), 8 μl Cas9 PLUS reagent (IDT), 16 μl CRISPRMAX reagent (Thermo Fisher) in 600 µl of Opti-MEM medium (Gibco) was prepared. 750,000 U-937 cells were washed with Opti-MEM medium (Gibco) and centrifuged at 300 x g for 5 min. The cell pellet was resuspended in the prepared solution and incubated at 37°C, 5% CO_2_. After a 24 h incubation, cells were centrifuged at 300 x g for 5 min, then resuspended in 400 μl flow buffer (HBSS, 1% FBS, 500 µM EDTA). The top 5% ATTO-550 positive cells were sorted on a BD FACSMelody™ Cell Sorter into four 96-well flat-bottom plates containing media at one cell per well. Approximately 2 weeks later, colonies were screened for Siglec-7 expression by flow cytometry using PE-conjugated Siglec-7 at 1:100 dilution (BioLegend) and Siglec-7^-/-^ clones were collected.

### Siglec-7 RNA Silencing of Primary Immune Cells

moDCs were transfected with two pre-designed small interfering RNA (siRNA) *Silencer Select* SIGLEC7 probes (ID# s25729 and s25730) or with the scramble siRNA (Invitrogen) with reverse transfection, as described previously (47). Briefly, 3 × 10^5^ moDC or moMϕ cells were incubated with the complex of two probes to a final 200 nM concentration or with the negative control (scramble) and 1% HiPerFect transfectant (Qiagen) in warm RPMI 1640 (non-supplemented) media in a 24-well plate for three days.

### Cytokine and Cell Surface Marker Analysis

moDCs or moMϕs or U-937-PMA (10^5^ cells) were cultured in 96-well plates in the Mercedes medium as described above and stimulated with PFA-fixed *F. nucleatum* ssp. at multiplicity of infection (MOI) of 50 or 5, *F. nucleatum* ssp.-derived LPS at 10 or 1 μg/ml, the *E. coli* O111:B4 control at 1 μg/ml, or OMVs at 5 x 10^7^ particles/ml for 18 h at 37°C. The cells were centrifuged at 510 x g for 3 min and the supernatant and pellet collected for analysis.

For cytokine analysis, human TNFα, IL-10, IL-8 production in the supernatant was quantified by ELISA (BioLegend) according to the manufacturer’s instructions.

For cell surface marker analysis, moDC or moMϕ pellets were first incubated with human Fc block (BioLegend) and then incubated with antibodies for 30 min at 4°C as follows: programmed death-ligand 1 (PD-L1)-PE at 1:50 dilution or CD80-PE at 1:100 dilution, CD86-Alexa488 at 1:200 dilution, isotype controls mouse IgG1-PE,κ at 1:100 (BioLegend) at 1:25 dilution (BioLegend) and with propidium iodide (PI) or 4′6-diamidino-2-phenylindole (DAPI) at 0.1 or 1 μg/ml for dead cell staining, respectively. The cells were then washed with DPBS supplemented with 1% BSA (FACS buffer) analysed by flow cytometry using Fortessa.

### Imaging Flow Cytometry

For counting bacteria, 10,000 events were collected and processed using the IDEAS (Amnis) software. Bacteria density ‘‘objects/ml’’ were selected in the bright field channel (M04) of the Aspect Ratio_M04 versus Area_M04 dot plot.

For internalisation assays, human cells (monocyte-derived or U-937-PMA) at 5 × 10^6^ cells/ml were incubated with 5 × 10^7^ FITC-stained *F. nucleatum* ssp. for 4 h. Cells were washed with FACS buffer and analysed by ImageStreamx Mk II (Amnis). Using the INSPIRE software, a total of 5,000 FITC stained cells were collected. The percentage of internalised bacteria were determined using the internalisation wizard with erode mask function at 7 number of pixels.

### Statistical Analyses

One-way ANOVA followed by Tukey’s test were used for multiple comparisons, t-test or two-way ANOVA were used for two-group comparisons, on Prism software (GraphPad). P < 0.05 was considered as statistically significant. *p < 0.05, **p < 0.01, ***p < 0.001, ****p < 0.0001, n.s., not statistically difference.

## Results

### *F. nucleatum* ssp. *animalis* Binding to Siglecs Revealed Specific Binding to Siglec-7

**Figure 1:**
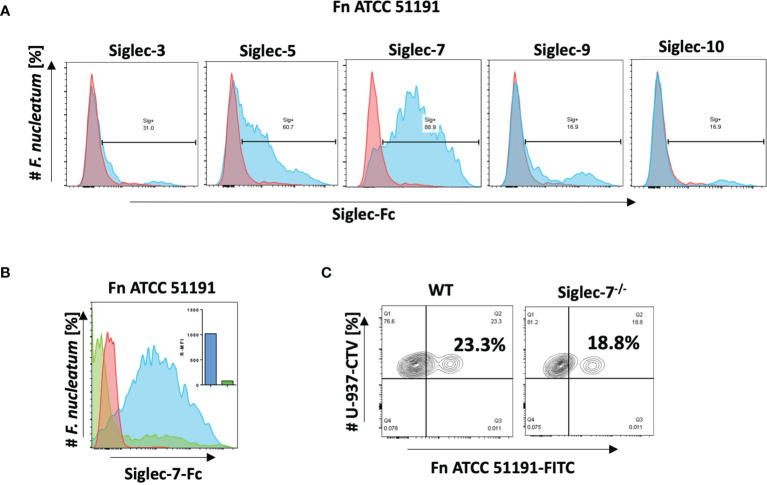
Binding of *F. nucleatum* ATCC 51191 to Siglecs using flow cytometry. **(A)** Binding of *F. nucleatum* to recombinant Siglec-Fc proteins (in blue). **(B)** Binding of *F. nucleatum* to Siglec-7 in the presence of GD3 inhibitor (in green) or untreated cells (in blue). **(C)** Binding of *F. nucleatum* to WT or Siglec-7^-/-^ U-937 cells. Bacteria incubated with α-Fc-PE Ab only was used as a control (in red). Fn, *F. nucleatum*.

The binding of *F. nucleatum* ATCC 51191 was first tested against a range of human recombinant CD33-related Siglec-Fc proteins including Siglec-3, Siglec-5, Siglec-7, Siglec-9 and -10 by flow cytometry. A clear shift in fluorescence was observed in the presence of Siglec-7 with approx. 90% of *F. nucleatum* population bound to Siglec-7, while 60% and 30% of *F. nucleatum* population bound to Siglec-5 and Siglec-3, respectively, and only 17% of the population bound to Siglec-9 and -10 (**Figure 1A**). To determine if the binding to Siglec-7 was glycan-mediated, inhibition binding assays were carried out in the presence of ganglioside GD3, a known Siglec-7 ligand (48). A significant decrease in Siglec-7 binding to *F. nucleatum* ATCC 51191 was observed in the presence of GD3, showing an approx. 92% reduction of the bacterial cell population bound to Siglec-7 (**Figure 1B**). This result suggests that Siglec-7 V-set domain is implicated in the binding between Siglec-7 and *F. nucleatum* ssp. To investigate whether the binding of *F. nucleatum* ATCC 51191 to Siglec-7 was mediated by sialic acid exposed on the bacterial cell surface, the bacterial cells were treated with neuraminidase A, a sialidase with broad specificity to (α2-3,6,8,9) sialylated linkages, cleaving linear and branched non-reducing terminal sialic acid residues from glycoconjugates. The sialidase pre-treatment only led to a small reduction in the binding of *F. nucleatum* to Siglec-7 as shown by flow cytometry (**Figure S1**), in agreement with the absence of sialic acid in *F. nucleatum* ATCC 51191 LPS (24). Next, we conducted binding assays between the monocytic cell line U-937 (wild type (WT) or Siglec-7^-/-^) and *F. nucleatum* ATCC 51191 by flow cytometry. Our results showed a reduction of *F. nucleatum* associated with U-937-Siglec-7^-/-^ cells as compared to WT cells (**Figure 1C**), supporting an interaction between *F. nucleatum* ssp. and Siglec-7 when expressed on the cell surface although other receptors may be involved in the interaction between *F. nucleatum* ATCC 51191 and U-937 cells.

### *F. nucleatum* Derived LPS or OMVs Bind to Siglec-7

**Figure 2:**
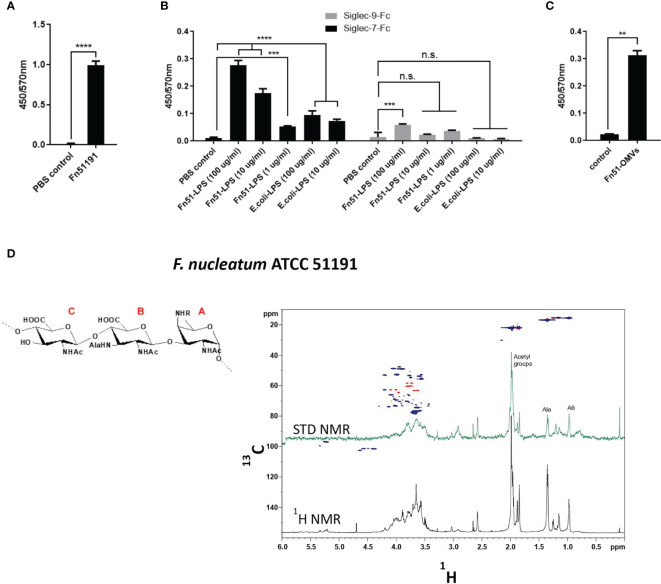
Binding of *F. nucleatum* ATCC 51191 cells, LPS or OMVs to Siglec-7-Fc. Immobilised *F. nucleatum* 51191 cells were tested for binding to **(A)** Siglec-7-Fc and the extracted LPS to **(B)** Siglec-7-Fc or Siglec-9-Fc by ELISA. **(C)** Immobilised OMVs extracted from *F. nucleatum* 51191 were tested for binding to Siglec-7-Fc by ELISA. PBS was used as control. Data shown are the mean of duplicates ± SD derived from one representative experiment reproduced in three independent experiments. Fn, *F. nucleatum*. **(D)** STD NMR analysis of the binding between Siglec-7 and partially depolymerised OPS from *F. nucleatum* ATCC 51191. The panel shows the superimposition of the reference ^1^H NMR spectrum (in black) and STD NMR spectrum (in green), the ^1^H-^13^C HSQC spectrum (blue/red) and the chemical structure of *F. nucleatum* OPS repeating units. Statistical analyses were performed by t-test (for panels 2Aand 2C) or one-way ANOVA followed by Tukey’s test. P < 0.05 was considered as statistically significant. **p < 0.01, ***p < 0.001, ****p < 0.0001, n.s., not statistically difference.

To determine the role of LPS in the binding of *F. nucleatum* ATCC 51191 to Siglec-7, LPS was extracted from *F. nucleatum* ATCC 51191 by the hot phenol/water method (41) and further purified by enzymatic digestion (41). The SDS-PAGE of the extracted *F. nucleatum*-derived LPS showed the typical LPS ladder-like pattern and lower average molecular weight distribution when compared to *E. coli* O127:B8 (**Figure S2**). Using an ELISA-type assay, we showed that the whole *F. nucleatum* cells and the derived LPS bound to Siglec-7-Fc (**Figures 2A, B**) while no binding of *F. nucleatum*-derived LPS was observed against Siglec-9-Fc used as a control (**Figure 2B**). To map the relevant positions of *F. nucleatum* LPS involved in the interaction with Siglec-7 and gain a first evaluation of the ligand epitopes, the partially depolymerised O-antigen chain (OPS) isolated from *F. nucleatum* ATCC 51191 was analysed by STD NMR (49) (**Figure 2D**). Interestingly, STD enhancements, together with changes in the relative intensity of STD signals with respect to the reference spectrum, were detected, clearly indicating that *F. nucleatum* ATCC 51191 OPS structure was recognised by and interacted with Siglec-7-Fc. Despite the significant overlapping of ligand resonances which impaired a detailed analysis of the protons involved in the recognition and binding process, the fingerprint of STD NMR spectrum allowed to identify the ligand regions in close contact with Siglec-7-Fc. *F. nucleatum* ATCC 51191 OPS contains a linear trisaccharide made up of glucosaminuronic (GlcNAcA and GlcNAc3NAlaA) and fucosamine (FucNAc4N) residues, [→4)‐β‐D‐GlcpNAcA‐(1→4)‐β‐D‐GlcpNAc3NAlaA‐(1→3)‐α‐D‐FucpNAc4NR‐(1→], with the N‐4 of the fucosamine partly acetylated (60 %). The analysis of signals in isolated regions of the spectrum, i.e., in the range between 0.8 – 1.5 ppm, demonstrated the contribution to the interaction from glucosaminuronic (GlcNAcA and GlcNAc3NAlaA) and fucosamine (FucNAc4N) residues. Therefore, STD NMR analysis confirmed binding of Siglec-7 to *F. nucleatum* ATCC 51191 OPS, even though it lacks nonulosonic acid residues (**Figure 2D**).

Next, we purified outer-membrane vesicles (OMVs) produced by *F. nucleatum* ATCC 51191 by density gradient ultracentrifugation, resulting in pure and spherical particles with a diameter range from 30 to 250 nm (**Figure S3A**). We showed by GC-MS that LPS is a main constituent (approximately in 60-70% mol/mol) of *F. nucleatum*-derived OMVs (**Figure S3B**). We then tested the ability of *F. nucleatum-*derived OMVs to bind to Siglec-7 (**Figure 2C**). *F. nucleatum-*derived OMVs from ATCC 51191 bound to Siglec-7 at levels comparable to LPS under the conditions tested (**Figure 2C**).

Together these data identified *F. nucleatum* LPS present on whole cells or OMVs as a new ligand to Siglec-7-Fc.

### *F. nucleatum* Modulates Immune Response in a Cell Subset Specific Manner

To investigate the impact of *F. nucleatum* ssp. on the host immune response, myeloid cells, moDCs and moMϕs, were generated from human blood, and stimulated with *F. nucleatum* ATCC 51191 or with *F. nucleatum* ATCC 51191-derived LPS and OMVs.

**Figure 3:**
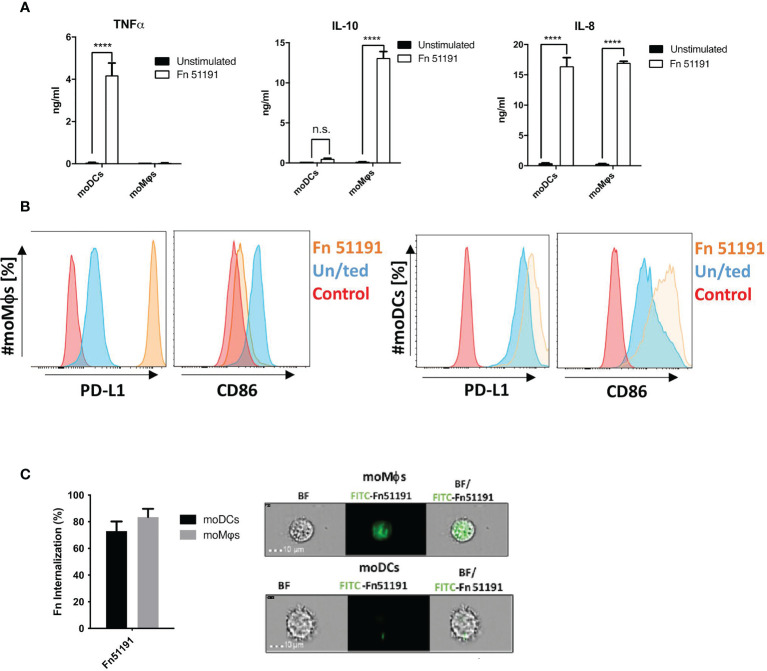
Effect of *F. nucleatum* ATCC 51191 on human myeloid cells. Analysis of **(A)** cytokine and **(B)** cell surface marker expression in moDCs or moMϕs by flow cytometry. Human cells were stimulated with *F. nucleatum* ATCC 51191 (in orange). Unstained cells (in red) and unstimulated (un/ted) cells (in blue) were used as controls. **(C)** Internalisation of *F. nucleatum* ATCC 51191 into moDCs or moMϕs. Images were taken with a 40X objective. For the cytokine quantification, data shown are the mean of triplicates ± SD derived from one representative experiment reproduced in three independent experiments. Statistical analyses were performed by one-way ANOVA followed by Tukey’s test. P < 0.05 was considered as statistically significant. ****p < 0.0001, n.s., not statistically difference.

*F. nucleatum* bacterial cells were shown to associate with the cell surface of moDCs or moMϕs as determined by imaging flow cytometry (**Figure S4**). Stimulation of moDCs with *F. nucleatum* at MOI 5 resulted in a marked increase in cytokine production of TNFα, IL-8 (p < 0.0001) (**Figure 3A**) and an induction of CD86 and PD-L1 as compared to the unstimulated control (**Figure 3B**). A different profile was observed with moMϕs, where stimulation with *F. nucleatum* led to a significant induction of IL-10 and IL-8 production (p < 0.0001), low levels (n.s.) of TNFα production (**Figure 3A**) and to the induction of PD-L1 and downregulation of CD86 cell surface markers as compared to the unstimulated control (**Figure 3B**). The results were dose-dependent, with a marked increase in cytokine production when cells were stimulated at MOI 50 as compared to MOI 5 (**Figure S5A**). This acquired moMϕ phenotype was also observed using the macrophage like cell line U-937 after *F. nucleatum* ATCC 51191 stimulation, leading to high IL-10 and low TNFα levels (**Figure S5B**). Consistent with these results, we showed, using imaging flow cytometry, that both moDCs and moMϕs were able to internalise *F. nucleatum* (**Figure 3C**), with moDCs showing approx. 10% less internalisation as compared to moMϕs (**Figure 3C**).

**Figure 4:**
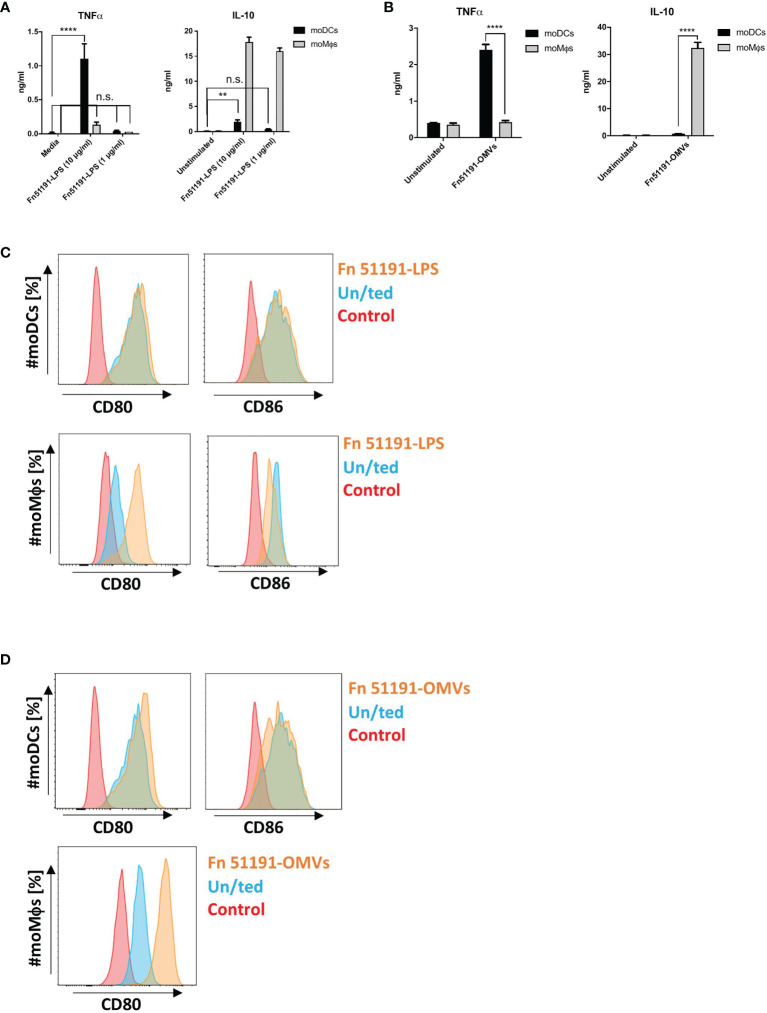
Effect of *F. nucleatum* ATCC 51191-derived LPS or OMVs on human myeloid cells. Analysis of **(A)** *F. nucleatum* LPS or **(B)** *F. nucleatum* OMVs on cytokine production and **(C)** *F. nucleatum* LPS or **(D)** *F. nucleatum* OMVs on cell surface marker expression. Unstimulated (un/ted) cells (in blue) and unstained cells (in red) were used as controls. Data shown are the mean of triplicates ± SD derived from one representative experiment reproduced in three independent experiments. Statistical analyses were performed by one-way ANOVA followed by Tukey’s test. P < 0.05 was considered as statistically significant. **p < 0.01, ****p < 0.0001, n.s., not statistically difference.

Next, we stimulated moDCs or moMϕs with *F. nucleatum* ATCC 51191-derived LPS or OMVs. In moDCs, treatment with OMVs or with LPS at 10 μg/ml but not 1 μg/ml induced TNFα production (**Figures 4A, B**). In moMϕs, stimulation OMVs or LPS (at both 10 or 1 μg/ml) showed significant induction of IL-10 at levels comparable to the whole bacteria (**Figures 4A, B**). When moMϕs or moDCs were treated with LPS or OMVs, there was an upregulation of the CD80 cell surface marker expression as compared to the unstimulated control, as showed with the whole bacteria. In moDCs, LPS stimulation led to an induction of CD86 expression (**Figure 4C**), as also observed with the whole bacteria, while stimulation with OMVs showed a reduction of CD86 expression compared to the unstimulated control (**Figure 4D**). Stimulation of moMϕs with LPS (at 10 or 1 μg/ml) showed a reduction of CD86 expression (**Figure 4C**), as also observed with the whole bacteria.

Overall, our results suggest that moDCs stimulated with *F. nucleatum* ATCC 51191 and derived components (OMVs and LPS) show a pro-inflammatory profile while *F. nucleatum*-treated moMϕs acquire a M2-phenotype which is associated with tumour progression (50).

### Siglec-7 Is Involved in *F. nucleatum*-Mediated Immune Response

**Figure 5:**
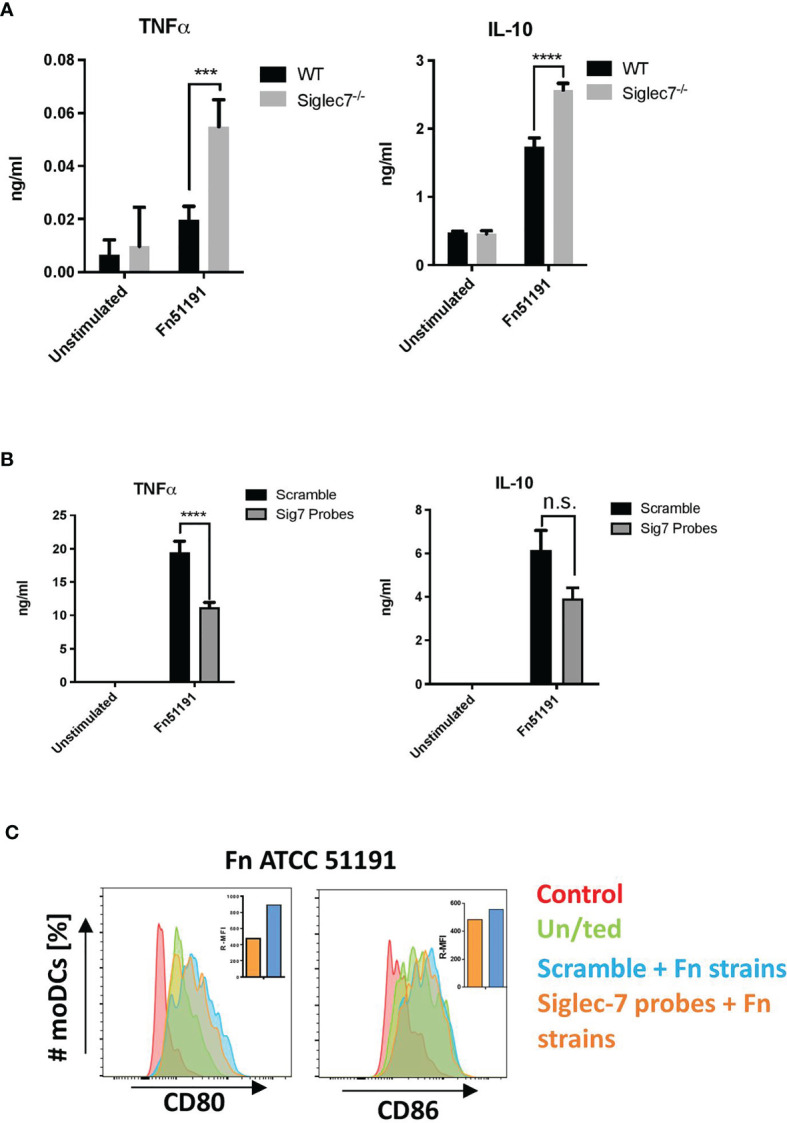
Effect of Siglec-7 on *F. nucleatum* ATCC 51191 interaction with human immune cells. **(A)** Cytokine production of U-937-PMA (WT or Siglec-7^-/-^) stimulated with *F. nucleatum* ATCC 51191. Bars represent the median values from 3 technical replicates. **(B)** Cytokine production and **(C)** cell surface marker expression of Siglec-7 silenced moDCs (in orange) or scramble control cells (in blue) stimulated with *F. nucleatum*. Unstimulated (un/ted) cells (in green) and unstained cells (in red) were used as controls. For the cytokine and internalisation analyses, data shown are the mean of triplicates ± SD and duplicated ± SD, respectively, derived from one representative experiment reproduced in three independent experiments. Statistical analyses were performed by two-way ANOVA followed by Tukey’s test. P < 0.05 was considered as statistically significant. ***p < 0.001, ****p < 0.0001, n.s., not statistically difference.

To obtain direct evidence of the contribution of Siglec-7 in *F. nucleatum* ATCC 51191 interaction with myeloid human cells, we used CRISPR-Cas9 editing to generate Siglec-7 deficient U-937 cells and assayed the effect of *F. nucleatum* stimulation on the immune response of differentiated U-937 WT or Siglec-7-deficient (Siglec-7^-/-^) cell lines (**Figure 5**). The expression of Siglec-7 in these cell lines was confirmed by flow cytometry (**Figure S6**). A significant increase (p < 0.01) in TNFα and IL-10 cytokine production was observed in *F. nucleatum* ssp.-stimulated U-937-Siglec-7^-/-^ as compared to WT cells (**Figure 5A**).

Next, we carried out silencing of Siglec-7 in primary moDCs and moMϕs by siRNA. Using flow cytometry, we confirmed that Siglec-7 was expressed on the cell surface of moDCs and moMϕs (**Figure S7A**), and that expression could be reduced by up to 40% in moDCs as compared to the scramble control (**Figure S7B**) while no significant reduction in expression could be achieved in moMϕs. We then analysed the cytokine profile and expression of cell surface markers following stimulation of silenced or scramble control moDCs with *F. nucleatum* ATCC 51191 at MOI 5 (**Figure 5B**). We showed that stimulation of Siglec-7 silenced moDCs with *F. nucleatum* produced statistically significant (p < 0.05) lower TNFα levels as compared to scramble moDCs (**Figure 5B**). No differences in cell surface marker expression were observed between *F. nucleatum*-stimulated Siglec-7 silenced or scramble moDCs (**Figure 5C**). Together these data suggest that Siglec-7 is involved in the induction of a pro-inflammatory response in moDCs by *F. nucleatum* ATCC 51191.

## Discussion

*F. nucleatum* is the most abundant bacterial species in the colorectal tumour microenvironment with *F. nucleatum ssp. animalis* ATCC 51191 being enriched in CRC tissues (11). *F. nucleatum* encodes an array of genes related to adhesion and invasion (51), enabling it to reside intracellularly in tumour cells (52), and, once there, potentially influencing tumorigenesis. Adhesion and invasion of *F. nucleatum* to epithelial cells are mediated by the Fap2 lectin and FadA adhesin expressed on the surface of *F. nucleatum*. The Fap2 lectin interacts with Gal-GalNAc glycans which are overexpressed on tumour cells (53), while the FadA adhesin, recognises and binds host surface components, such as vascular endothelial cadherin (54) and epithelial cadherin (16). *F. nucleatum* FadA expression was also shown to be upregulated in CRC tissue (16). Interaction of FadA with E-cadherin triggers the expression of oncogenes, such as c-MYC and inflammatory genes, through the β-catenin cascade and the upregulation of annenix A1 (16, 17). However, the receptors involved in the interaction between *F. nucleatum* ssp. and immune cells remain largely unknown.

Here, we showed that *F. nucleatum* ssp*. animalis* ATCC 51191 interacts with Siglec-7 expressed by immune cells and that binding is LPS-mediated. Human NK cells, macrophages, dendritic cells constitutively express Siglec-7, and the colonic lamina propria monocytes and macrophages represent the major Siglec-7 positive populations (55). Extracellularly, Siglec-7 has a sialic acid-binding V-set domain which we demonstrated was implicated in the binding to *F. nucleatum*. Siglec-7 has been shown to bind to the sialylated ganglioside GD3 (56), and N-linked disialyl Lewis^a^ in the normal colonic epithelium (55). At the molecular level, Siglec-7 has been reported to bind to terminal sialic acid moieties with diverse underlying glycan structures. We recently uncovered the LPS structure of *F. nucleatum* ATCC 51191, revealing a novel sugar repeating unit in the O-antigen structure [→4)-β-D-Glc*p*NAcA-(1→4)-β-D-GlcpNAc3NAlaA-(1→3)-α-D-Fuc*p*NAc4NR-(1→], (R= Acetylated 60%), and a bis-phosphorylated hexa-acylated lipid A moiety (24). It therefore likely that the LPS glycans other than sialic acid moieties may contribute to the binding of *F. nucleatum* ATCC 51191 to Siglec-7, consistent with the results of the sialidase treatment. This was further confirmed by STD NMR, showing that the OPS extracted from *F. nucleatum* ATCC 51191 was recognised by Siglec-7, revealing new ligand epitopes not restricted to nonulosonic acids (neuraminic acid and fusaminic acid). The discovery that *F. nucleatum* LPS is a ligand for immune checkpoint receptors like Siglec-7 opens up new blockade strategies and studies are in progress to gain further structural insights into the broad ligand specificity of Siglec-7 towards the bacterial glycan structures revealed in this work.

We showed that *F. nucleatum* ATCC 51191 induced a pro-inflammatory profile in moDCs and a tumour associated profile in macrophages (moMϕs and U-937 cells) and that Siglec-7 contributed to these cell-specific responses using Siglec-7 RNA-silenced moDCs and Siglec-7 deficient U-937 cells. In macrophages, *F. nucleatum* ATCC 51191 induced the expression of IL-10, IL-8 cytokines and PD-L1 marker and a downregulation of CD86 cell surface marker expression, characteristic of macrophage type 2 (M2) polarisation (12, 13). These results are in agreement with previous studies showing an infiltration of M2-macrophages in *F. nucleatum* ssp. positive clinical CRC specimens (57) and a M2 acquired phenotype in macrophage-like cell lines stimulated with *F. nucleatum* ATCC 10953 (13), and *F. nucleatum* ATCC 25586 (58). A recent study showed that Siglec-7 and -9 induce the polarisation of monocytes into a tumour-associated macrophage (TAM) phenotype and the induction of tumour-associated cell surface markers such as PD-L1 (59). The moDC response to *F. nucleatum* ATCC 51191 suggests that Siglec-7 contributes to the pro-inflammatory response. These cell-specific phenotypes could be recapitulated using *F. nucleatum* derived OMVs or LPS, implicating LPS as a ligand of the interaction with Siglec-7. The interaction of *F. nucleatum* derived LPS has been reported with TLR-4 leading to polarisation of macrophages, a process that is associated with tumour cell proliferation and metastasis (13). Our findings that LPS and OMVs influence innate immune cell responses is supported by recent studies showing that *F. nucleatum* OMVs can trigger inflammation of human intestinal epithelial cells (IECs) (60), by promoting NF-κB activation in a TLR-2-dependent manner (61).

Siglec-7 can bind to a range of human cell types (such as basophils, eosinophils, NK cells and splenocytes), illustrating its role of ‘self’ recognition (62). The response we observed upon interaction with *F. nucleatum* differs from the canonical inhibition of immune activity observed between immune cells and cancer cells (25) but is consistent with *in vivo* work using ApcMin/+ model reporting that *F. nucleatum* induced expression of the genes encoding several pro-inflammatory cytokines, including TNFα, IL-6, IL-8 and IL-1β (12) which mirrors human RNA-seq data from patients bearing high *F. nucleatum* loads in their colorectal tumours (12). To our knowledge, only one study, using Siglec-7 silencing approach in monocytes, also showed association of Siglec-7 with pro-inflammatory cytokine production, upon interaction with yeast particles in a sialic acid-independent manner (63). These differences in cell immune response could be attributed to the mode of recognition, the nature of the interactions (cell-cell or cell-microbe), or the heterogeneity in pattern recognition receptor (PRR) expression in the different cell subsets used in the *in vitro* studies. Indeed, other PRRs may act synergistically with Siglec-7 to contribute to a distinct immune response. For example, TLR-4, a toll-like receptor with an intracellular activation motif has been shown to establish a direct interaction with Siglecs including Siglec-7 (64). Therefore, since TLR-4 is expressed in U-937 (65) and moDCs (66), our findings could be the result of a synergetic effect between Siglec-7 and TLR-4. This interaction could also contribute to the capacity of *F. nucleatum* ssp. to promote chemoresistance of CRC by inhibition of cancer cell apoptosis (8–10). A recent *in vivo* study using humanized immunocompetent mice, showed that Siglec-7 and -9 could be potential targets to enhance anti-tumour immunity (36). In the future, it will be interesting to study the effect of *F. nucleatum*-Siglec-7 interaction *in vivo*, using humanised immunocompetent murine model, as Siglec-E, the closest murine homolog of Siglec-7, does not recognise *F. nucleatum* ssp. (data not shown), consistent with the lack of direct homology between murine and human Siglecs (67).

It was recently reported that Siglec−7 is expressed in macrophages in CRC tissue from patients and that high levels of Siglec−7 expression in tumour tissues are associated with shorter overall survival in patients treated with immunotherapy for metastatic CRC (68). Mirroring this, an independent human study reported that patients with high relative abundance of *F. nucleatum* in tumour tissues compared to matched control tissues have a higher incidence of regional lymph node metastases (69). These studies support the translation of our findings to humans. The interaction of *F. nucleatum* ssp. with Siglec-7, leading to a pro-inflammatory microenvironment, provides a mechanism underpinning these associations in patients and initial evidence that blocking this interaction may be a potential strategy to alleviate the progression of *F. nucleatum* associated CRC.

In summary, our results reporting LPS-mediated interaction of *F. nucleatum* and derived OMVs with Siglec-7, add a new dimension in our understanding of the role of Siglecs in cancer progression. Given the role of *F. nucleatum* in influencing CRC tumorigenesis and response to cancer treatment, there is significant interest in developing strategies that target *F. nucleatum*, preferably in the tumour tissue. However, antimicrobial strategies are limited due to concerns about antibiotic resistance and the mutualistic role of *F. nucleatum* in the oral cavity and other mucosal sites of humans (70). Targeted glycan interventions to displace Siglec-7-*F. nucleatum* interactions could prove an effective way of improving current approaches for the treatment of cancer by targeting *F. nucleatum* in the tumour environment and without compromising the rest of the gut microbiome or inducing antimicrobial resistance.

## Data Availability Statement

The original contributions presented in the study are included in the article/**Supplementary Material**. Further inquiries can be directed to the corresponding author.

## Ethics Statement

Blood collection in this study was approved by the Faculty of Medicine and Health Sciences Research Ethics Committee REC reference number 2013/2014 -14HT (University of East Anglia).

## Author Contributions

Conceptualization, DL and NJ. Experimental work, DL, PG-V, KM, and RM. Resources and materials, NJ, PC, MM, CH, and KB. Writing—original draft preparation, NJ and DL. Review and editing, DL, PC, CC, and NJ. Supervision, NJ, AS, MM, CC, and KB. Funding acquisition, NJ. All authors contributed to the article and approved the submitted version.

## Conflict of Interest

The authors declare that the research was conducted in the absence of any commercial or financial relationships that could be construed as a potential conflict of interest.

## Publisher’s Note

All claims expressed in this article are solely those of the authors and do not necessarily represent those of their affiliated organizations, or those of the publisher, the editors and the reviewers. Any product that may be evaluated in this article, or claim that may be made by its manufacturer, is not guaranteed or endorsed by the publisher.
