# Supplementary material for: Siglec-7 Mediates Immunomodulation by Colorectal Cancer-Associated Fusobacterium nucleatum ssp. animalis
Source: Front Immunol. 2021 Oct 1;12:744184. doi: 10.3389/fimmu.2021.744184 (PMC8517482; doi:10.3389/fimmu.2021.744184)
Supplement: Supplementary file 1 [file DataSheet_1.pdf]

Supplementary Material

Supplementary Figures

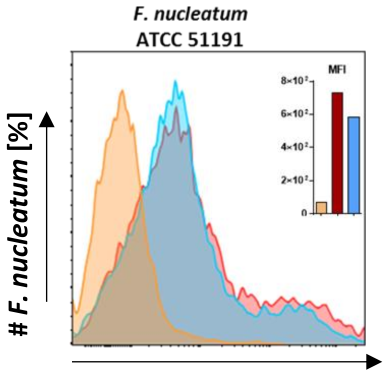

Supplementary Figure 1. Effect of sialidase treatment on *F. nucleatum* ATCC 51191.- Siglec-7 interaction. Flow cytometry was used to analyse the binding between Siglec-7-Fc and sialidase-treated *F. nucleatum* (in blue) or untreated bacteria (in red). Fn, *F. nucleatum*; MFI, mean fluorescence intensity. Bacteria incubated with antibody only (in orange) were used as a control.

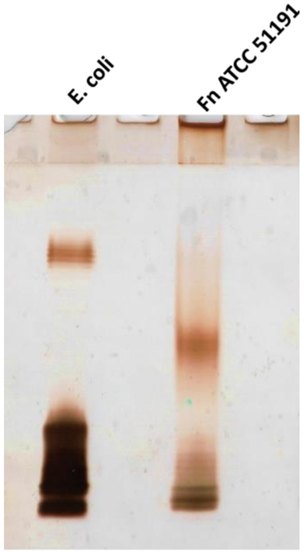

Supplementary Figure 2. Electrophoresis analysis of *F. nucleatum* ATCC 51191 LPS. LPS from *F. nucleatum* was analysed on a silver-stained SDS-PAGE 12% and compared to LPS from *E. coli* O127:B8.

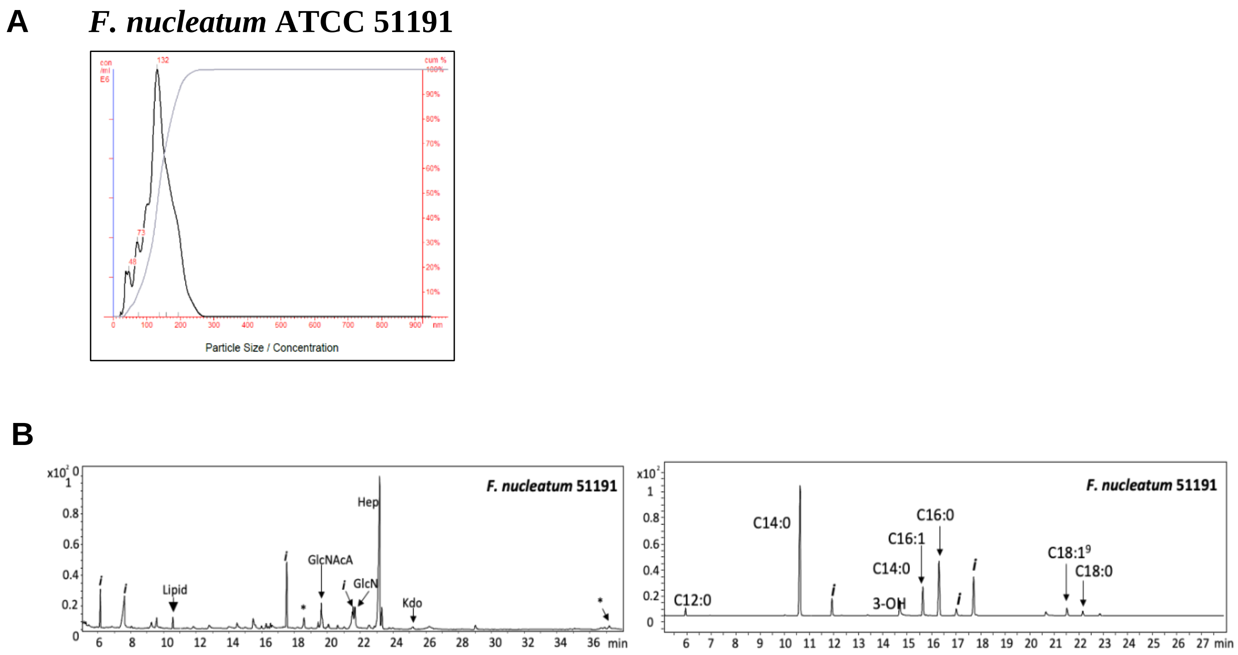

Supplementary Figure 3. Analysis of *F. nucleatum* ATCC 51191 -derived OMV particles. A. *F. nucleatum* OMV particle size was analysed by NanoSight. The main peak corresponds to the mode of the population. **B.** Purified OMVs from *F. nucleatum* ATCC 51191 were used for Acetylated O-methyl glycoside (left) and total fatty acid composition analysis by methanolysis (right) using GC-MS. Man: mannose, Gal: galactose, Glc: glucose, GlcN: glucosamine, Hep: L-*glycero*-D-*manno*-heptopyranose, Kdo: 3-deoxy-D-*manno*-oct-2-ulopyranosonic acid, Neu5Ac: N-Acetylneuraminic acid, Simple and saturated fatty acids are named as Cx:0, x being the total number of carbons of the fatty acid; C-3 hydroxylated fatty acids as Cx:0 (3-OH). *unknown monosaccharide, *i*: impurity.

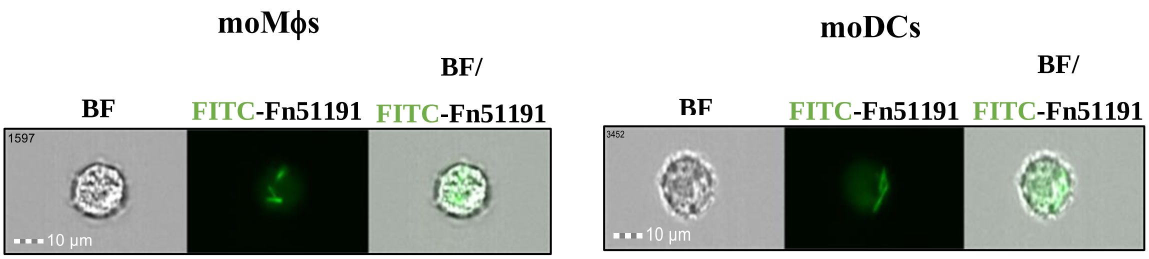

Supplementary Figure 4. *F. nucleatum* ATCC 51191 association with human myeloid cells. Association of FITC-labelled *F. nucleatum* (in green) with moDCs or moMϕs was determined by imaging flow cytometry. Images were taken with 40X objective. Fn, *F. nucleatum*.

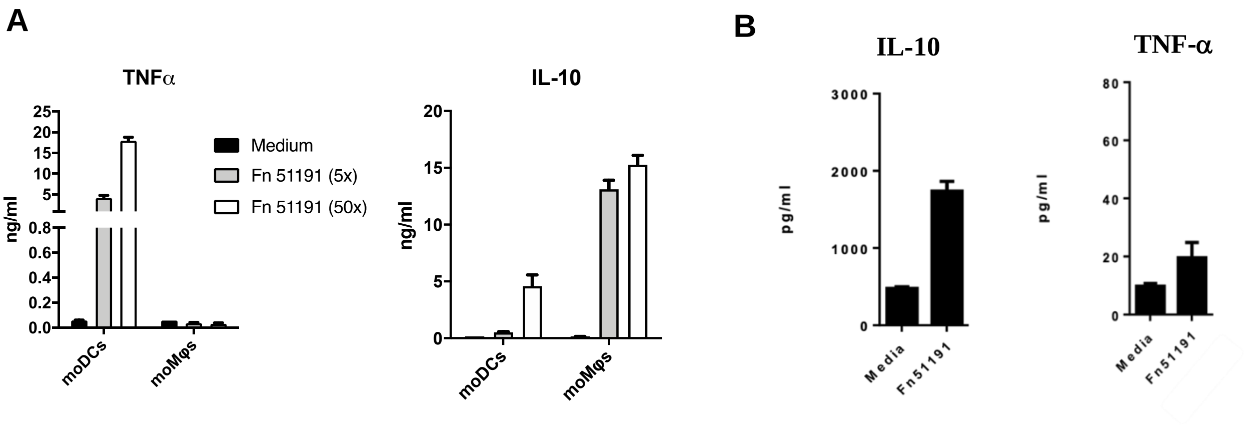

Supplementary Figure 5. Effect of *F. nucleatum* ATCC 51191 on cytokine production by immune cells. A. Cytokine analysis of the primary human cells (moDCs or moMϕs) stimulated with *F. nucleatum* ATCC 51191 at MOI of 5 or 50 as colour coded. Medium alone (in red) was used as a control. **B.** Cytokine analysis of the macrophage-differentiated monocytic cell line U-937 stimulated with *F. nucleatum* ATCC 51191 at MOI 5. Data shown are the mean of triplicates from one representative experiment reproduced in two or three independent experiments. Fn, *F.*

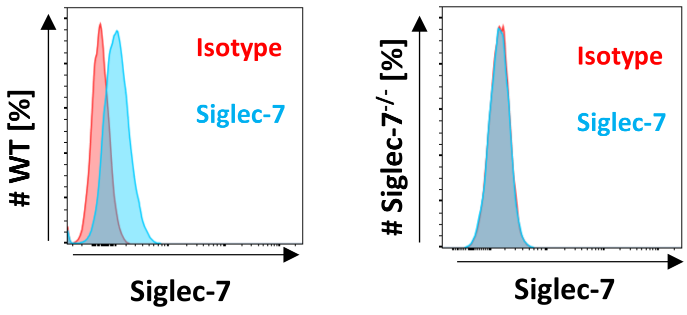

Supplementary Figure 6. Siglec-7 expression on U-937 WT, and Siglec-7$^{-/-}$ (generated by CRISPR-Cas9) cells by flow cytometry. Expression of Siglec-7 (in blue) or isotype (in red).

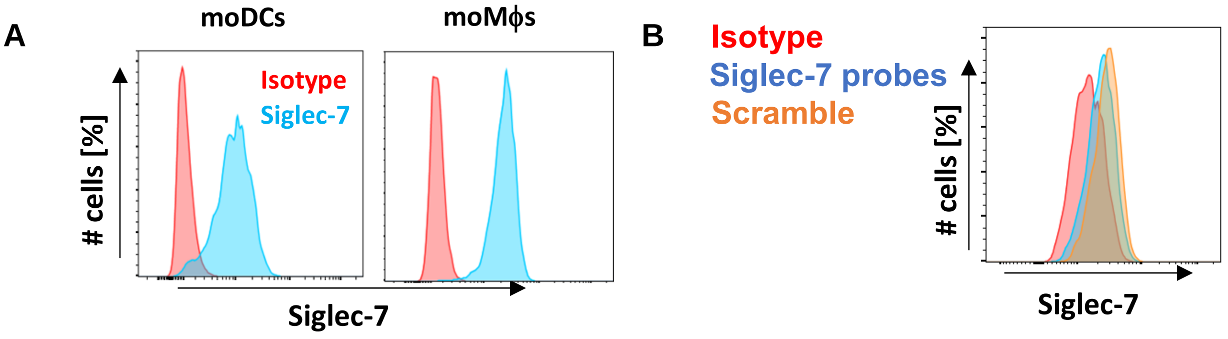

Supplementary Figure 7. Siglec-7 expression in myeloid cells by flow cytometry. Siglec-7 expression was determined on **A.** Human moDCs, moMϕs cell surface (in blue) **B.** Analysis of Siglec-7 RNA silenced (in blue) or scramble control (in orange) moDCs. The isotype was used as control (in red).
